# Evaluation of glycemic status and subclinical atherosclerosis in familial hypercholesterolemia subjects with or without LDL receptor mutation

**DOI:** 10.1186/s12933-025-02683-y

**Published:** 2025-03-20

**Authors:** Francesco Di Giacomo Barbagallo, Giosiana Bosco, Maurizio Di Marco, Sabrina Scilletta, Nicoletta Miano, Marco Musmeci, Marina Martedì, Ana M. González-Lleó, Daiana Ibarretxe, Ernestina Marianna De Francesco, Roberta Malaguarnera, Antonino Di Pino, Luís Masana, Francesco Purrello, Salvatore Piro, Roberto Scicali

**Affiliations:** 1 Department of Clinical and Experimental Medicine, Internal Medicine, Garibaldi Hospital, University of Catania, Via Palermo 636, 95122 Catania, Italy; 2 Unitat Medicina Vascular I Metabolisme, Unitat de Recerca en Lìpids I Arterioslcerosi, Hospital Universitari Sant Joan, Universitat Rovira I Virgili, IISPV, Reus, Spain; 3 Department of Medicine and Surgery, “Kore” University of Enna, Enna, Italy

**Keywords:** Familial hypercholesterolemia, Low-density lipoprotein receptor, Glycemic status, Subclinical atherosclerosis, Cardiovascular risk

## Abstract

**Background:**

Familial hypercholesterolemia (FH) is a genetic condition characterized by elevated LDL-C and increased cardiovascular risk. Beyond LDL-C levels, the impact of genotype on glucose homeostasis has not been well evaluated. We aimed to evaluate the impact of genotype on glycemic status and on atherosclerotic injury in FH subjects.

**Methods:**

We conducted a cross-sectional study on 322 FH subjects not on lipid-lowering therapy and without history of cardiovascular disease. Biochemical and genetic analyses as well as vascular profile assessment were obtained from all subjects. The study population was divided into two groups according to genotype: LDL receptor (LDLR) group and non-LDLR (NLDLR) group.

**Results:**

The LDLR group exhibited a higher prevalence of low glycemic status (LGS) than the NLDLR group (44.1% vs. 26%, *p* < 0.01), whereas a high glycemic status (HGS) was more prevalent in the NLDLR group compared with LDLR group (74% vs. 55.9%, *p* < 0.01). The NLDLR group exhibited a higher prevalence of peripheral atherosclerotic plaques than the LDLR group (93.4% vs. 73%, *p* < 0.05), while coronary artery calcification (CAC) presence was more prevalent in the LDLR group compared with the NLDLR group (74.7% vs. 48%, *p* < 0.01). In a secondary analysis the study population was stratified into three groups based on LDLR genotype: NLDLR, LDLR defective, LDLR null groups. The prevalence of LGS progressively increased from the NLDLR to the LDLR null group, while HGS showed an inverse trend (*p* for trend < 0.05). Peripheral atherosclerotic plaque prevalence decreased from the NLDLR to the LDLR null group (*p* for trend < 0.05), while CAC prevalence increased progressively in the three groups (*p* for trend < 0.01). Logistic regression analysis showed that FH groups with an LDLR mutation were inversely associated with HGS (*p* for both < 0.01) and the LDLR null group exhibited the strongest association.

**Conclusions:**

FH subjects with NLDLR mutations exhibited a worse glycemic profile, while null LDLR mutations showed the strongest inverse association with HGS. The integrations of genetic, lipid and glucose data could be useful to better identify the metabolic profile and the atherosclerosis distribution in FH subjects.

**Graphical abstract:**

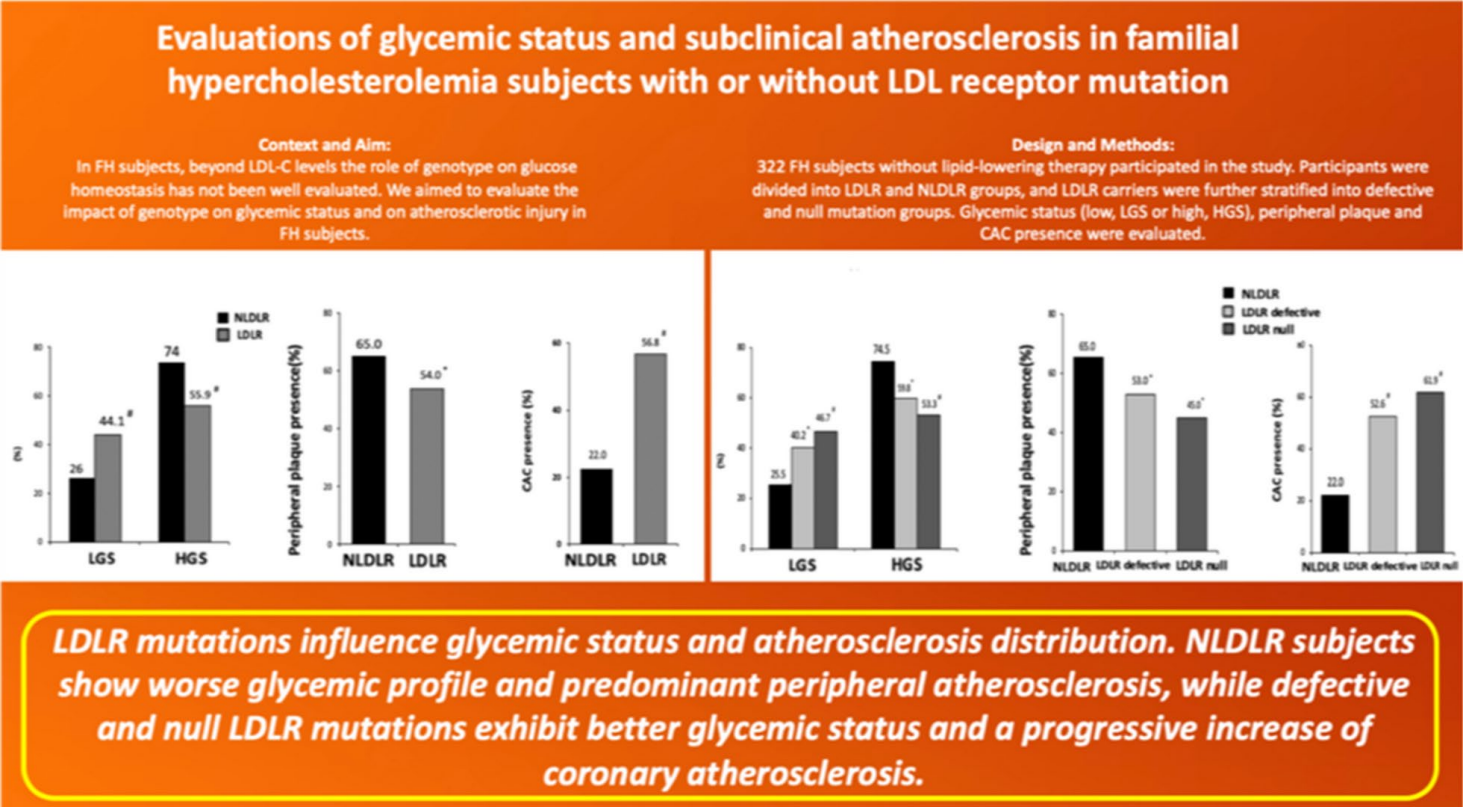

**Research insights:**

**What is currently known about this topic?:**

Familial hypercholesterolemia (FH) is characterized by elevated LDL-C levels. LDLR null mutations protected pancreatic β-cells from cholesterol accumulation. NGS has improved FH diagnosis by analysis of all genes implicated in the lipid disorder.

**What is the key research question?:**

What is the impact of FH genotype (monogenic with or without LDLR mutation/polygenic) on glycemic status?

**What is new?:**

FH population was characterized by a heterogeneous glycemic profile according to LDLR mutation. LDL-C and plasma glucose could modulate the distribution of subclinical atherosclerosis.

**How might this studyinfluence clinical practice?:**

Genetic, lipid, glucose data could better identify the metabolic and atherosclerotic profiles in FH.

## Background

Familial hypercholesterolemia (FH) is the most common monogenic lipid disorder characterized by lifelong exposure to high levels of low-density lipoprotein cholesterol (LDL-C) and by an increased risk of premature atherosclerotic cardiovascular disease (ASCVD) [1]. In FH subjects different mutations in genes encoding for LDL-C metabolism key proteins promote a decreased LDL-C uptake and thus an increased plasma LDL-C concentration [2]; of these, the majority are present in the LDL receptor (LDLR) accounting for 85–90% of cases, while a low prevalence of genetic variants have been reported in apolipoprotein B (ApoB), proprotein convertase sublisin kexin type 9 (PCSK9) or apolipoprotein E (ApoE) [3]. To date, over 3,000 mutations have been identified in the LDLR gene and they are mainly classified into null or defective mutations that result in a complete or partial impairment of receptor activity, respectively [4].

While the causal role of LDL-C in atherosclerotic injury was largely evaluated, in FH subjects increasing attention has been focused on the impact of genetic variants in glucose homeostasis [5].

Several studies showed that LDLR null mutations protected pancreatic β-cells from cholesterol accumulation and thus a reduced risk of insulin resistance have been observed in these subjects [6, 7]; however, defective LDLR mutations or mutations in other FH genes could contribute to a cholesterol-induced metabolic dysfunction in FH subjects with a residual/normal LDLR activity [8]. In this context, it could be hypothesized that LDLR may play an important role in glucose homeostasis. Moreover, an impaired glucose metabolism could promote the accumulation of atherogenic lipoproteins leading to the progression of vascular damage [9, 10].

In FH subjects an early and intensive lipid-lowering therapy (LLT) is needed to reduce both LDL-C plasma levels and the risk of cardiovascular events [11]; of these, statin treatment is the cornerstone of LDL-C reduction and should be initiated as soon as possible, even during childhood [12]. However, due to the increased expression of LDLR on hepatocytes, statins may increase the risk of diabetes development, especially in predisposed individuals receiving high-intensity statin treatments; moreover, the “diabetogenic” effect of statins could also be influenced by the type of genetic condition in FH subjects [13, 14]. In the last few years the advances of novel genetic diagnostic strategies such as next-generation sequencing (NGS) has improved the ability to diagnose FH by a comprehensive analysis of all genes implicated in the lipid disorder, including those associated with a polygenic condition [15]. No data on glucose homeostasis and vascular damage have been reported in FH subjects stratified according to a monogenic or polygenic condition.

In this study we aimed to investigate the impact of genotype on glycemic status and on the atherosclerotic injury distribution in a cohort of FH subjects.

## Methods

### Study design and population

This was a cross-sectional observational study involving individuals with a probable or defined clinical diagnosis of FH (Dutch Lipid Clinical Network score ≥ 6) who had undergone genetic analysis [2, 16] from July 2016 to September 2024. All participants were enrolled from the University Hospital of Catania, Italy, which is a tertiary center for the screening, diagnosis and management of familial dyslipidemias. All subjects were aged between 18 and 70 years and were not on LLT at the time of enrollment; moreover, all subjects were free of ASCVD, hematopoietic disorders, malignancies and/or treatment with chemotherapy, acute infections, chronic inflammatory status and glucocorticoid therapy within the past three months. After a 12-h fast, all participants underwent a physical examination and review of their clinical history as well as biochemical analyses and vascular profile evaluation by assessments of coronary artery calcium (CAC) score and carotid and femoral plaques. Monogenic FH was defined as the presence of a genetic variant in one of the following genes: LDLR, Apolipoprotein B (ApoB), Proprotein Convertase Subtilisin/Kexin type 9 (PCSK9), or Apolipoprotein E (ApoE), while the diagnosis of recessive hypercholesterolemia (ARH) was confirmed by the presence of two genetic variants in low density lipoprotein receptor adaptor protein 1 (LDLRAP1) [17]. In subjects without a monogenic variant, polygenic FH was defined as the presence of a polygenic LDL-C score > 0.73 [18]. Subclinical atherosclerosis (SA) was defined as a CAC score > 0 and/or presence of carotid and/or femoral plaques [19–21].

Arterial hypertension was defined as brachial blood pressure (BP) ≥ 140 mm Hg (systolic) and/or 90 mm Hg (diastolic) on at least two different occasions, or if the subject was on antihypertensive therapy [22]. Body weight and height were measured, and body mass index (BMI) was calculated as weight divided by the squared value of height (kg/m^2^) [23]. Type 2 diabetes (T2D) was defined as a fasting plasma glucose (FPG) ≥ 126 mg/dL on two consecutive readings and/or glycated hemoglobin (HbA1c) ≥ 6.5% or the use of anti-diabetic medications [24]. According to the median values of FPG and HbA1c, low glycemic status (LGS) was defined as FPG < 88 mg/dL and/or HbA1c < 5.5% and high glycemic status (HGS) was defined as FPG ≥ 88 mg/dL and/or HbA1c ≥ 5.5%. Smoking habits were divided into either current smoking (defined as a minimum of one cigarette in the last month) or not [25]. Participants were stratified into two groups according to genotype: FH subjects with LDLR mutation (LDLR group, 222 subjects) and FH subjects without LDL receptor mutation (NLDLR group, 100 subjects).

The study was approved by the local ethics committee in accordance with the ethical standards of the institutional and national research committees and with the 1964 Declaration of Helsinki and its later amendments or comparable ethical standards. Informed consent was obtained from each subject enrolled in the study.

### Biochemical analyses

FPG was measured with the glucose oxidase method. Serum total cholesterol (TC), triglycerides (TG), high-density lipoprotein cholesterol (HDL-C), and high sensitivity protein C reactive (hs-CRP) were assessed by currently available enzymatic methods. LDL-C was calculated using the Friedewald formula. Non-HDL cholesterol (Non-HDL-C) and TG/HDL were derived from baseline values. The triglyceride-glucose (TyG) index was calculated according the following formula: Ln [(TGxFPG)/2)] [26]. ApoB and Apolipoprotein A1 (ApoA1) were evaluated with a nephelometer assay (Siemens AG Healthcare Sector, Erlangen, Germany). Levels of lipoprotein(a) [Lp(a)] were measured with the latex agglutination immunoassay [27]. HbA1c was measured with high-performance liquid chromatography using a National Glycohemoglobin Standardization Program and standardized to the Diabetes Control and Complications Trial assay reference. Chromatography was performed using a certified automated analyzer (HPLC; HLC-723G7 hemoglobin HPLC analyzer; Tosoh Corp.; normal range 4.25–5.9% [23–41 mmol/mol]) [28].

### CAC assessment

Each patient underwent a multi-detector computed tomography (CT) scan (Definition Flash, Siemens, Erlangen, Germany), with a total radiation exposure ranging from 1 to 3 mSv. CAC was assessed using the Agatston scoring method [29]. Coronary imaging was performed without contrast, using the high-resolution mode of the ultrafast CT scanner with a scan time of 100 ms, a slice thickness of 3 mm, ECG gating, and breath-hold technique. A total of 20 contiguous slices (covering 60 mm) were acquired, starting from the lower margin of the pulmonary artery bifurcation.

The presence and extent of coronary calcification were evaluated at each slice level. A calcified lesion was defined as having a CT density of at least 130 Hounsfield units and a minimum area of 1 mm^2^. The CAC score was calculated as the product of the calcified plaque area and the maximum lesion density, classified from 1 to 4 based on Hounsfield units. All CT scans were analyzed in a specialized central reading facility and reviewed by a senior cardiovascular radiologist blinded to the patients’ medical history. CAC presence was defined as a score greater than 0.

### Carotid and femoral plaque assessments

Ultrasound assessments of the carotid and femoral arteries were conducted using an ACUSON Sequoia system equipped with an 8 MHz transducer, following previously established protocols [30]. Carotid evaluations were performed over a 1 cm segment of the distal common carotid artery, located 1 cm proximal to the dilation of the carotid bulb, and a 1 cm segment of the carotid bifurcation, positioned 1 cm proximal to the flow divider. Similarly, femoral measurements focused on the common femoral artery, specifically 1 cm proximal to its bifurcation.

For both the right and left carotid and femoral arteries, three longitudinal sections were captured. Plaques in the carotid and femoral arteries were identified based on an intima-media thickness (IMT) exceeding 1.5 mm. Peripheral atherosclerosis was defined as the presence of carotid and/or femoral plaques. All examinations were conducted by a single operator who was blinded to patient details.

### Statistical analysis

The distributional characteristics of each variable, including normality, were assessed by the Kolmogorov–Smirnov test. Data are reported as mean ± standard deviation (SD) for continuous parametric and median (interquartile range-IQR) for continuous non-parametric variables and as frequency (percentage) for categorical variables. When necessary, continuous non-parametric variables (TG, Lp(a), hs-CRP) were logarithmically transformed for statistical analysis to reduce skewness. The Chi square (χ2) test was used for categorical variables. To test differences in clinical and biochemical characteristics between the groups Student’s t-test was used. In order to evaluate the role of genotype on glycemic status, we performed a logistic regression analysis adjusted for age, waist circumference, BMI, LDL-C, systolic BP, diastolic BP; moreover, the estimation of variance inflation factor due to covariates was < 2. In a secondary analysis, the study population was stratified into three groups according to LDLR genotype: FH subjects without LDLR mutation (NLDLR group, 100 individuals), FH subjects with LDLR defective mutation (LDLR defective group, 137 individuals), FH subjects with LDLR null mutation (LDLR null group, 85 individuals). Finally, a χ2 test was performed to assess the distributions of glycemic status, peripheral plaque presence and CAC presence in the three groups.

All statistical analyses were performed using IBM SPSS Statistics for Windows version 23. For all tests, a *p* < 0.05 was considered significant.

## Results

**Fig. 1:**
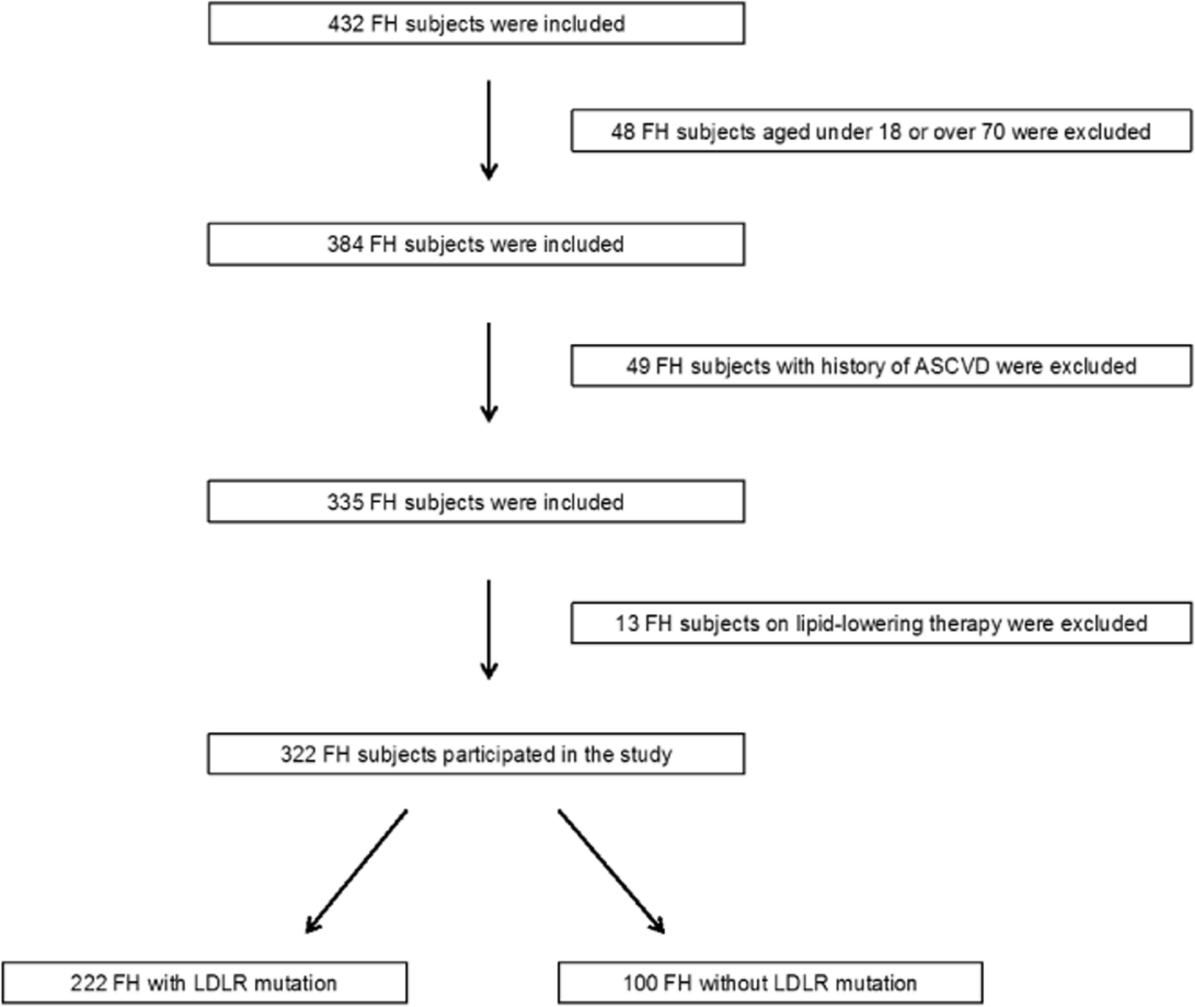
Enrollment flowchart of the study population. FH = familial hypercholesterolemia, ASCVD = atherosclerotic cardiovascular disease, LDLR = low-density lipoprotein receptor

A total of 432 subjects were evaluated; of these, 322 subjects satisfied the inclusion criteria and participated in the study (Fig. 1).

The characteristics of the study population are presented in Table 1. A monogenic FH was present in 77% of subjects and the LDLR mutation was the most common genetic variant, while 23.0% of subjects exhibited polygenic FH; moreover, the majority of LDLR mutations were defective, while 38.3% of these were classified as null mutations. The prevalence of SA in the study population was 61.5%; of these, CAC and peripheral plaque presences were 74.7% and 93.4%, respectively. Furthermore, a CAC score > 0 as well as the presence of peripheral plaque were observed in more than a third of these subjects.

**Table 1:** Characteristics of the study population

N	322
FH genotype	
Monogenic FH, n (%)	248 (77.0)
LDLR, n (%)	222 (89.5)
*LDLR defective, n (%)*	137 (61.7)
*LDLR null, n (%)*	85 (38.3)
ApoB, n (%)	19 (7.7)
PCSK9, n (%)	3 (1.2)
ApoE, n (%)	4 (1.6)
Polygenic FH, n (%)	74 (23.0)
Monogenic FH phenotype	
Heterozygous, n (%)	266 (100)
Vascular profile	
Subclinical atherosclerosis, n (%)	198 (61.5)
CAC > 0, n (%)	148 (74.7)
Peripheral plaque, n (%)	185 (93.4)
CAC + Peripheral plaque, n (%)	73 (36.9)

Table 2 shows the general characteristics of the study population stratified into two groups according to genotype. Age as well as the prevalence of men and BMI were similar between the two groups. While the prevalence of T2D was similar between the two groups, FPG as well as HbA1c were higher in the NLDLR group compared with the LDLR group (for FPG 95.91 ± 6.21 vs. 88.23 ± 5.16, *p* < 0.05; for HbA1c 5.61 ± 0.47 vs. 5.50 ± 0.39, *p* < 0.05). TC as well as LDL-C and Non-HDL-C were significantly higher in the LDLR group than the NLDLR group (for TC 369.85 ± 22.97 vs. 325.08 ± 21.71; for LDL-C 270.27 ± 25.51 vs. 225.39 ± 24.86; for Non-HDL-C 158.61 ± 30.33 vs. 128.59 ± 25.71, *p* value for all < 0.001), while an increase of the TG plasma level and TyG was observed in the NLDLR group more than the LDLR group (for TG 101 [77–139] vs. 81 [64–104]; for TyG 8.43 ± 0.42 vs. 7.20 ± 0.41, for both *p* < 0.001).

**Table 2:** General characteristics of the study population stratified according to genotype

	LDLR group	NLDLR group	*p* value between two groups
Demographic characteristics			
*N*	222	100	–
Age, years	55.09 ± 8.05	56.42 ± 7.63	0.16
Men, n (%)	121 (54.5)	48 (48)	0.19
Body mass index, kg/m^2^	25.65 ± 3.66	25.88 ± 3.52	0.58
Glucose profile			
Type 2 diabetes, n (%)	12 (5.4)	7 (7.0)	0.54
FPG, mg/dL	88.23 ± 5.16	95.91 ± 6.21	< 0.05
HbA1c, %	5.50 ± 0.39	5.61 ± 0.47	< 0.05
Lipid profile			
TC, mg/dL	369.85 ± 22.97	325.08 ± 21.71	< 0.001
HDL-C, mg/dL	52.28 ± 6.83	54.08 ± 8.59	0.12
Triglycerides, mg/dL	81 (64–104)	101 (77–139)	< 0.001
LDL-C, mg/dL	270.27 ± 25.51	225.39 ± 24.86	< 0.001
Non-HDL-C, mg/dL	158.61 ± 30.33	128.59 ± 25.71	< 0.001
TG/HDL	1.82 ± 1.14	1.99 ± 0.84	0.87
TyG	7.20 ± 0.41	8.43 ± 0.42	< 0.001
ApoB, mg/dL	140.36 ± 5.32	138.04 ± 5.03	0.80
ApoAI, m g/dL	137.92 ± 10.62	141.05 ± 10.67	0.21
ApoB to ApoAI ratio	1.06 ± 0.44	0.95 ± 0.24	0.18
Lp(a), mg/dL	20.9 (10.4–40.4)	19.9 (10.1–59.5)	0.12
Risk factors			
Systolic BP, mmHg	118.98 ± 9.51	120.15 ± 9.46	0.47
Diastolic BP, mmHg	70.88 ± 9.27	72.78 ± 9.82	0.13
Smoking, n (%)	74 (33.3)	33 (33)	0.86
hs-CRP, mg/dL	0.09 (0.05–0.21)	0.12 (0.05–0.24)	0.60

**Fig. 2:**
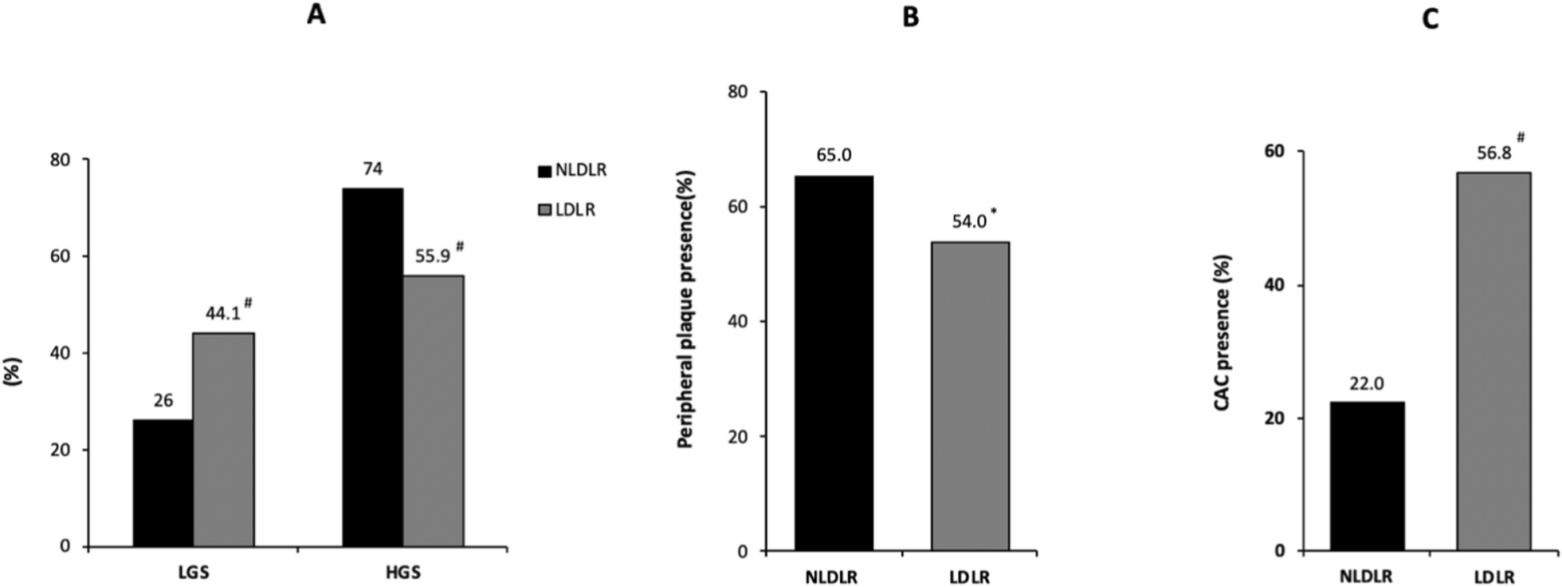
Distribution of glycemic status, peripheral plaque and CAC presence in the study population stratified according to genotype. **A** shows the distribution of glycemic status into the groups; **B** shows the distribution of peripheral plaque presence into the groups; **C** shows the distribution of CAC presence into the groups. LGS = low glycemic status, HGS = high glycemic status, NLDLR = non-low-density lipoprotein receptor, LDLR = low-density lipoprotein receptor, CAC = coronary artery calcium. ^*^p < 0.05 versus NLDLR, ^#^*p* < 0.01 versus NLDLR

Figure 2 illustrates the distributions of glycemic status, peripheral plaque and CAC presence in the study population stratified into two groups according to genotype. While the LDLR group exhibited a higher prevalence of subjects with LGS than the NLDLR group, the percentage of subjects with HGS was higher in the NLDLR group than in the LDLR group (44.1% vs. 26% and 74% vs. 55.9%, respectively, for both *p* < 0.01; Fig. 2A). The proportion of patients with peripheral plaque was greater in the NLDLR group compared with the LDLR group (65% vs. 54%, *p* < 0.05; Fig. 2B) while the prevalence of subjects with CAC was significantly higher in the LDLR group than in the NLDLR group (56.8% vs. 22%, *p* < 0.01; Fig. 2C).

**Fig. 3:**
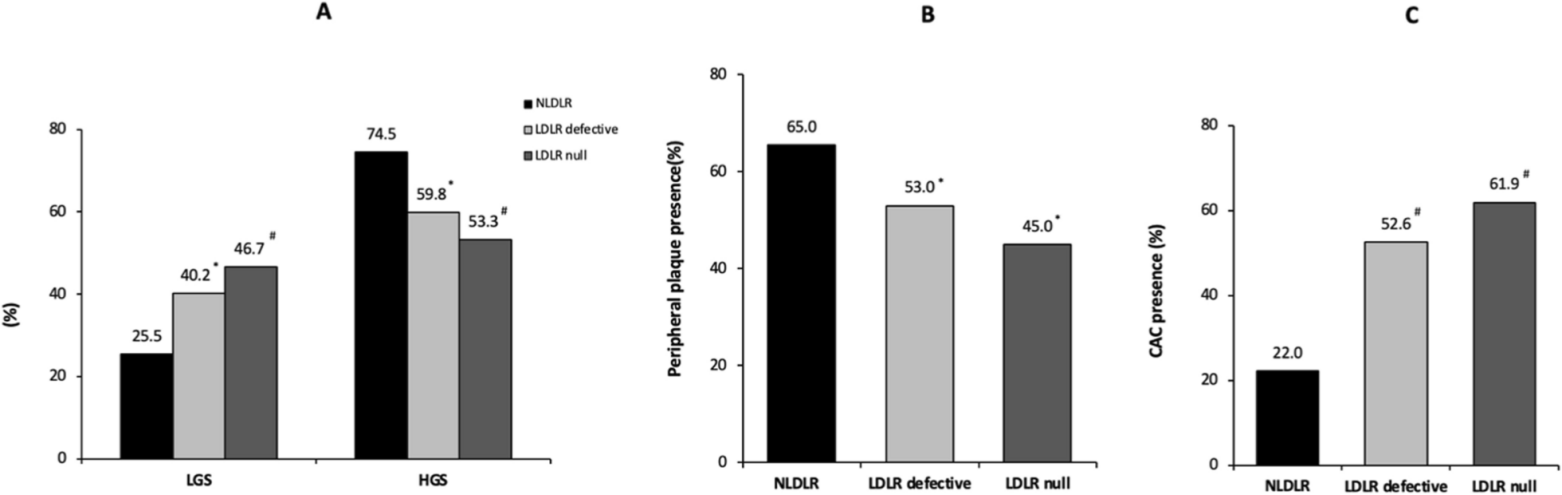
Distribution of glycemic status, peripheral plaque and CAC presence in the study population stratified according to LDLR genotype. **A** shows the distribution of glycemic status in the groups; **B** shows the distribution of peripheral plaque presence in the groups; **C** shows the distribution of CAC presence into the groups. LGS = low glycemic status, HGS = high glycemic status, NLDLR = non-low-density lipoprotein receptor, LDLR = low-density lipoprotein receptor, CAC = coronary artery calcium. ^*^p < 0.05 versus NLDLR, ^#^*p* < 0.01 versus NLDLR

Figure 3 shows the distribution of glycemic status, peripheral plaque and CAC presence in the study population stratified into three groups according to LDLR genotype. While the percentage of subjects with LGS progressively increased from the NLDLR group to the LDLR null group, (*p* for trend < 0.05), the prevalence of patients with HGS decreased from the NLDLR group to the LDLR null group (*p* for trend < 0.05; Fig. 3A). The prevalence of peripheral plaque decreased from the NLDLR group to the LDLR null group (*p* for trend < 0.05; Fig. 3B), while the proportion of patients with CAC significantly increased across the same groups (*p* for trend < 0.05; Fig. 3C). Multivariate logistic regression analysis showed that FH with an LDLR mutation were inversely associated with a high glycemic status (for both* p* < 0.01) and the LDLR null group exhibited the strongest association **(**Table 3**)**.

**Table 3:** Logistic regression of glycemic status in the Study Population

Dependent variable	High glycemic status	
Independent variable	Multivariate ORs (95% CIs)	
	Model	*P* value
NLDLR, n 100	1 (reference)	–
LDLR defective, n 137	0.46 (0.263–0.804)	< 0.01
LDLR null, n 85	0.374 (0.194–0.719)	< 0.01
Age, years	1.124 (1.089–1.161)	< 0.001
Waist circumference, cm	1.103 (1.025–1.186)	< 0.01
BMI, kg/m^2^	1.266 (0.194–0.719)	< 0.05
LDL-C, mg/dL	1.001 (0.996–1.005)	0.57
Systolic BP, mmHg	1.013 (1.003–1.057)	0.28
Dyastolic BP, mmHg	1.006 (0.998–1.012)	0.32

Logistic regression model was used to estimate ORs and 95% CIs

Model was adjusted for age, waist circumference, BMI, LDL-C, Systolic BP, Diastolic BP

*NLDLR* Non-low-density lipoprotein receptor, *LDLR* low-density lipoprotein receptor, *BMI* body mass index, *LDL-C* low-density lipoprotein cholesterol, *BP* blood pressure

## Discussion

In this study we investigated the impact of genotype on glycemic homeostasis and atherosclerotic injury in FH subjects not on lipid lowering therapies; to the best of our knowledge, this is the first study exploring the interactions of genetic profile and glycemic status with atherosclerotic damage in this population. We found that the NLDLR group exhibited higher levels of FPG and HbA1c than the LDLR group; moreover, the proportion of subjects with HGS was higher in the NLDLR group than in the LDLR defective and null groups. Finally, FH with LDLR defective and null mutations were inversely associated with high glycemic status and the LDLR null group exhibited the strongest association. Our results are in line with previous studies that evaluated the impact of FH genotype on pancreatic β-cell function and glucose metabolism [31–33]; these studies highlight the critical role of LDLR in pancreatic β-cell physiology. In fact, Da Dalt et al. demonstrated that PCSK9 KO mice exhibited reduced insulin secretion, glucose intolerance and an increased cholesteryl ester accumulation in β-cells compared with wild-type mice and these alterations were reversed in PCSK9/LDLR double KO mice, suggesting that LDLR mediates cholesterol-induced β-cell dysfunction and insulin resistance by promoting intracellular cholesterol accumulation [32]; moreover, Paquette et al. showed that β-cells are particularly susceptible to cholesterol overload due to their limited capacity for cholesterol efflux [31]. Excess intracellular cholesterol disrupts mitochondrial ATP production, increases reactive oxygen species (ROS) generation, and promotes oxidative stress, ultimately leading to insulin secretory dysfunction and β-cell apoptosis [31, 33]. Thus, the lack of LDL-C internalization in FH subjects may mitigate these pathogenic processes preserving β-cell function and reducing the risk of metabolic dysfunction and insulin resistance [34].

In this context, in a large cohort study Besseling et al. previously reported a lower percentage of diabetes in FH subjects than the unaffected relatives; moreover, null mutation carriers exhibited the lowest prevalence of diabetes. In line with these findings, in our study we reported a different glycemic status according to FH genotype; furthermore, we also demonstrated a relationship between genotype, glucose metabolism and atherosclerosis distribution [35]. Null LDLR mutations completely abolish the receptor function reducing intracellular LDL-C transport and protecting pancreatic β-cells from cholesterol accumulation [8]. In contrast, FH individuals with defective LDLR mutations or without LDLR mutations are characterized by a residual receptor activity that could be able to promote intracellular cholesterol transport leading to impaired glucose homeostasis [6].

As concerns the vascular profile, in our study we found different atherosclerotic damage distributions based on LDLR genotype; in this context, we showed that the prevalence of subjects with CAC was higher in LDLR null and defective groups compared with the NLDLR group. This may be mainly explained by an increased lifelong exposure to high LDL-C levels in FH with LDLR mutation than without that promotes the accumulation of oxidized LDL in the arterial walls as well as the inflammatory cascade progression and thus the atherosclerotic process in the coronary arteries [36, 37]. However, in our study the percentage of FH subjects with peripheral atherosclerosis was higher in the NLDLR group than LDLR groups. Our findings are in line with previous studies indicating that impaired glucose homeostasis accelerates atherosclerotic progression, particularly in peripheral arterial districts [38, 39]. In fact, DeFronzo et al. showed that insulin resistance promotes endothelial dysfunction by reducing NO bioavailability and activating pro-inflammatory signaling pathways, primarily via MAPK/ERK; moreover, Janus et al. demonstrated that insulin resistance alters endothelial homeostasis by impairing NO synthesis and exacerbating oxidative stress, thereby promoting atherogenesis [39]. Furthermore, Kolluru et al. reported that chronic hyperglycemia and insulin resistance upregulate adhesion molecules (ICAM-1, VCAM-1) facilitating monocyte/macrophage recruitment and contributing to peripheral atherosclerosis [40]. These mechanisms may underlie the higher prevalence of peripheral plaques observed in the NLDLR group, reinforcing the hypothesis that insulin resistance plays a key role in shaping the distribution of atherosclerotic burden.

In this context, increased plasma glucose levels promote the formation of advanced glycation end-products (AGEs), which bind to their receptors (RAGE) on endothelial cells initiating a cascade of pathological events that induce oxidative stress, endothelial dysfunction and activation of vascular smooth muscle cells [41, 42]. These processes contribute to lipid accumulation, vascular remodeling and arterial stiffness exacerbating the progression and destabilization of atherosclerotic plaques in the peripheral districts. Moreover, hyperglycemia and insulin resistance stimulate systemic inflammation by the release of pro-inflammatory cytokines such as interleukin-6 (IL-6) and tumor necrosis factor-alpha (TNF-α) aggravating vascular damage [43, 44].

In recent years, TyG has emerged as a reliable biomarker linking lipid metabolism alterations to insulin resistance and atherosclerotic burden. TyG reflects the interplay between glucose and lipid metabolisms and it has been proposed as an early biomarker of subclinical atherosclerosis and cardiovascular risk in dysmetabolic patients [45]. In our study, we showed that TyG was significantly higher in the NLDLR group than in the LDLR group, indicating a greater severity of insulin resistance in subjects without LDL receptor mutations. Moreover, we found a higher prevalence of peripheral plaques observed in the NLDLR group compared with LDLR group, supporting the role of insulin resistance in shaping atherosclerotic burden distribution. Our findings were in line with previous large cohort studies [46, 47]; in fact, Liu et al. reported that TyG was independently associated with an increased prevalence of peripheral artery disease, reinforcing the role of insulin resistance in non-coronary vascular involvement. Moreover, Ding et al. found that TyG correlated with increased arterial stiffness and endothelial dysfunction in individuals with impaired glucose homeostasis.

There are several limitations to our study. First, because of its cross-sectional design, the impact of genotype on the changes in glycemic status and vascular profile over time cannot be established.Moreover, the study did not evaluate environmental and behavioral factors, such as dietary habits and physical activity, which may have influenced metabolic and atherosclerotic profiles. Moreover, our study population did not undergo CT angiography, despite its ability to offer detailed plaque characterization and the identification of vulnerable plaques [48]. However, we used less invasive and widely accessible diagnostic methodologies, such as the Agatston CAC score, a well-established marker of subclinical atherosclerosis and a strong predictor of cardiovascular events both in the general population and in FH subjects [49]. Furthermore, the immune system parameters as well as specific inflammatory biomarkers such as AGEs, IL-6 or TNF-α were not evaluated. Further prospective studies are needed to assess circulating inflammatory biomarker levels and their relationships with genotype, glycemic homeostasis and the progression of atherosclerotic injury in FH subjects.

## Conclusions

In conclusion, in our study FH subjects with NLDLR mutations exhibited a worse glycemic profile than the LDLR group, while null LDLR mutation FH showed the strongest inverse association with a high glycemic status. To better characterize the phenotype of the FH population we reported a heterogeneous glycemic profile according to the presence or not of LDLR mutation; moreover, it may be possible that according to genotype the interaction of LDL-C levels and plasma glucose appears to play a role in modulating the distribution of subclinical atherosclerosis. Taking into consideration these findings, the integrations of genetic, lipid and glucose data could be useful to better identify the metabolic profile and the atherosclerosis distribution in FH subjects.

Further prospective studies are needed to assess circulating inflammatory biomarker levels and their relationships with genotype, glycemic homeostasis and the progression of atherosclerotic injury in FH subjects.

## Data Availability

No datasets were generated or analysed during the current study.

## Abbreviations

ApoA1: Apolipoprotein A1
ApoB: Apolipoprotein B
ApoE: Apolipoprotein E
AGEs: Advanced glycation end-products
ASCVD: Atherosclerotic cardiovascular disease
BMI: Body mass index
BP: Blood pressure
CAC: Coronary artery calcium
CT: Computed tomography
FH: Familial hypercholesterolemia
FPG: Fasting plasma glucose
HbA1c: Glycated hemoglobin
HDL-C: High-density lipoprotein cholesterol
HGS: High glycemic status
IMT: Intima-media thickness
IL-6: Interleukin-6
Lp(a): Lipoprotein(a)
LDL-C: Low-density lipoprotein cholesterol
LDLR: Low-density lipoprotein receptor
LDLRAP1: Low density lipoprotein receptor adaptor protein 1
LGS: Low glycemic status
LLT: Lipid-lowering therapy
NGS: Next-generation sequencing
PCSK9: Proprotein convertase sublisin kexin type 9
SA: Subclinical atherosclerosis
T2D: Type 2 diabetes
TC: Total cholesterol
TG: Triglycerides
TyG: Triglyceride-glucose index
TNF-α: Tumor necrosis factor-alpha
